# Photodynamic bone stabilization for traumatic and pathologic fractures: a systematic review of utilization, complications, and patient-reported outcomes

**DOI:** 10.1007/s00590-024-03833-w

**Published:** 2024-02-20

**Authors:** Kevin Y. Zhu, Ryan McNassor, Christian J. Hecht II, Robert J. Burkhart, Lukas M. Nystrom, Atul F. Kamath

**Affiliations:** 1 Department of Orthopedic Surgery, Center for Hip Preservation, Orthopaedic and Rheumatologic Institute, Cleveland Clinic Foundation, 9500 Euclid Ave, Cleveland, OH 44195 USA; 2 Department of Orthopedic Surgery, University Hospitals, Cleveland, OH 44195 USA

**Keywords:** Photodynamic bone stabilization, Pathologic fracture, Traumatic fracture, Minimally invasive surgery, Patient-reported outcomes, Complications

## Abstract

**Introduction:**

The photodynamic bone stabilization system (PBSS) was was developed in 2010, and in 2018 gained FDA approval in the United States. Given its relative novelty, our analysis sought to analyze the available literature exploring the indications, outcomes, and complications of the PBSS.

**Methods:**

We performed a systematic review (PROSPERO registration of study protocol: CRD42022363065, October 8th, 2022). PubMed, EBSCOHost, and Google Scholar electronic databases were queried to identify articles evaluating PBSS in the treatment of pathologic or traumatic fractures between January 1 2010 and 15 October 2022. The quality of the included studies was assessed using the Methodological Index for Nonrandomized Studies tool.

**Results:**

Our initial search yielded 326 publications, which were then screened for appropriate studies that aligned with the purpose of our review. A total of thirteen studies, comprising seven case series, four case reports, and two cohort studies. The total sample size of the included studies consisted of 345 patients, with 242 females (70%) and 103 males (30%). The implants were most commonly utilized in the humerus (41%), radius (12%), and metacarpal (12%). The most common complications were related to broken implants (5%) and dislocation (1%). Most studies reported complete fracture healing and return of full strength and range of motion.

**Conclusion:**

Despite being a relatively novel technology, PBSS appears to be a viable option for fracture stabilization. Most studies included in our analysis reported complete fracture healing and return of function with minimal complications.

## Introduction

A photodynamic bone stabilization system (PBSS) was developed in 2010 for the treatment of fractures, especially pathologic, osteoporotic, and impending fractures [1, 2]. The system is based on a light curable monomer within a balloon catheter that can provide longitudinal and rotational stability by conforming to the shape of the intramedullary canal upon exposure to blue light to form a rigid implant in the target bone [3]. Currently, this technology has obtained approval from the United States Food and Drug Administration (FDA) for investigational device usage within the United States. Additionally, it is currently being utilized in clinical settings in several European countries, such as Germany, Austria, Switzerland, and Italy [2, 4–6].

**Fig. 1:**
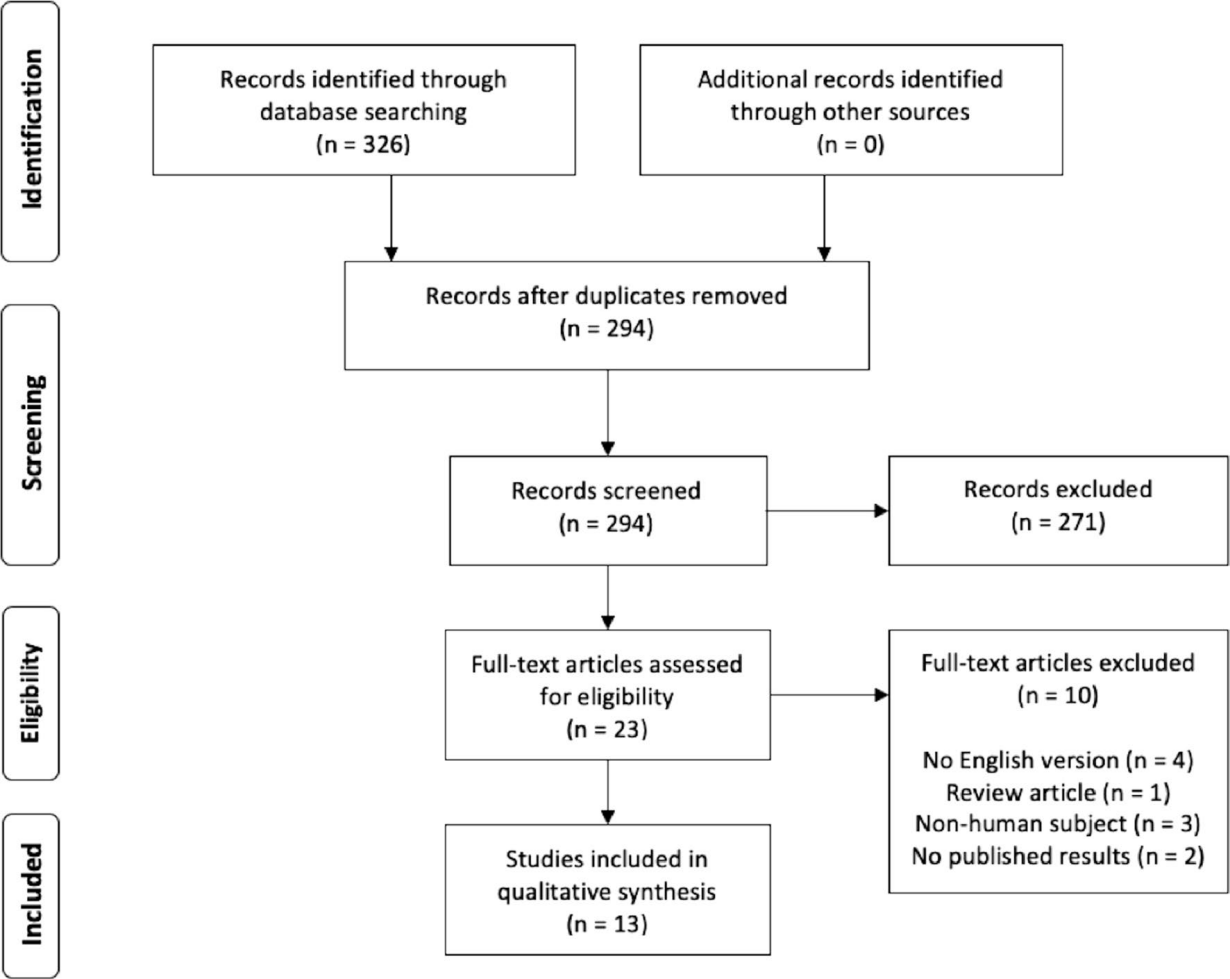
PRISMA flowchart outlining the study selection process

While traditional methods of fracture stabilization such as intramedullary nails and plate fixation are capable of providing sufficient stabilization in healthy bone, they are not specifically designed or suitable for addressing fragility fractures that arise due to weakened, osteoporotic bone. The PBSS offers several advantages in the treatment of fractures and impending fractures. It allows for minimally invasive surgery through a percutaneous incision with preservation of endosteal blood supply and decreases the disruption of muscular attachments [7]. The system employs a light-cured, on-demand polymerizing mechanism that, unlike bone cement that hardens rapidly, affords the surgeon the flexibility and adequate time to achieve the appropriate reduction of the fracture before polymerization takes place (Fig. 1). Several studies have found this system effective in treating fractures of the tibia, fibula, and humerus, exhibiting minimal complications and high rates of fracture healing [2, 7–9].

To better understand the current interventions and overall efficacy, we conducted a systematic review to evaluate the utilization, complications, patient-reported outcomes, and radiographic outcomes of PBSS.

## Methods

This review was conducted according to the Preferred Reporting Items for Systematic Reviews and Meta-Analysis guidelines (PRISMA) [10]. The study protocol was registered prospectively with PROSPERO registration of the study protocol was CRD42022363065, October 8th, 2022.

### Literature search

The PubMed, EBSCO Host, and Google Scholar electronic databases were queried with the following keywords or MeSH terms in combination with ‘AND’ or ‘OR’ Boolean operators: ‘photodynamic bone stabilization [MeSH]’; ‘Illuminoss’; ‘bone stabilization system’; ‘intramedullary stabilization’; ‘intramedullary osteosynthesis’; ‘Fracture [MeSH]; ‘Osteolyses’; ‘Osteolysis’; ‘Indications’; ‘advantages’; ‘limitations;’ ‘limits;’ ‘outcomes’; ‘follow-up’; ‘management’; ‘safety’; ‘recovery’; and ‘treatment’ to identify all studies that evaluated PBSS in the treatment of pathologic or traumatic fractures between 1 January 2010 and 15 October 2022.

Articles were included if the following criteria were met: (1) full-text articles in English are available, (2) the study evaluated the PBSS in the treatment of a fracture(s), (3) the study used live human subjects, and (4) identified an outcome (e.g., survival, complications, radiologic outcomes, functional outcomes). Review articles, cadaver studies, and animal studies were excluded.

### Study selection

**Fig. 2:**
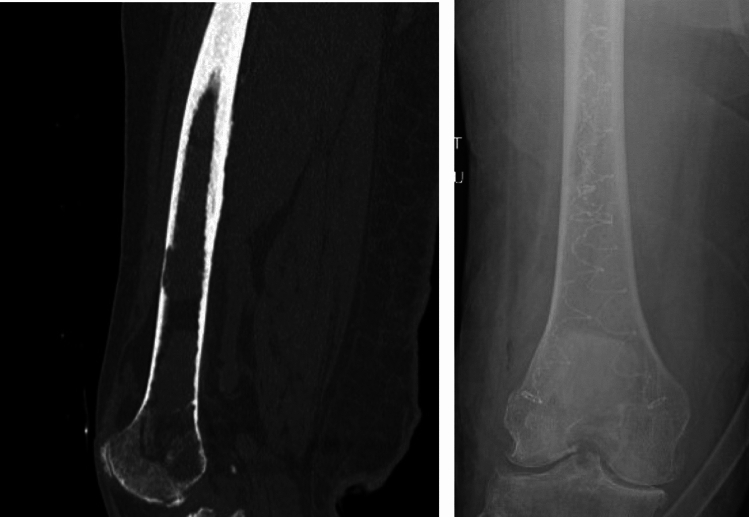
Impending pathologic fracture of the right distal femur metadiaphysis treated with double balloon implants

Two reviewers independently assessed the eligibility of each article included in the review. In the case of disagreement, a third reviewer was consulted to reach consensus. The initial query yielded 326 publications, which were then screened for relevant studies for the purposes of our review. After removing duplicates and reviewing each abstract for relevancy 23 were selected for full text review. Of these, 13 fulfilled our inclusion and exclusion criteria. A thorough review of each reference’s list did not yield any additional studies. The selection process is shown in Fig. 2.

### Data collection

A collaborative spreadsheet, arranged by two reviewers before starting, facilitated data extraction. Study characteristics and outcomes were extracted from each study and verified by a second reviewer. Characteristics extracted included publication year, medical journal, study design, level of evidence, total study population, age, sex, number of fractures, fracture type (traumatic or pathologic), and fracture location. In addition, outcomes related to complications, radiographic, and functional findings were recorded. Complications were further classified as intraoperative complications (any complication reported during the operative procedure), postoperative device-related complications, and non-device-related complications. Radiographic and functional findings were reported descriptively due to the heterogeneity of the fractures being analyzed in the included studies.

### Risk of bias in individual studies

The risk of bias was assessed by two independent reviewers using the MINORS tool. This is a validated assessment tool that assigns a value from 0 to 24 to comparative studies based on 12 criteria related to study design, outcomes assessed, and follow-up. Higher scores indicate a higher quality of study. Across these domains, each item is graded with a 2 when adequately reported, 1 when reported but inadequate, and 0 if not reported. Any discrepancies in the grading were resolved by discussion and consultation with a third reviewer. Case reports were not eligible for MINORS and excluded from the risk of bias assessment [11].

## Results

### Included studies

There were thirteen studies, comprising seven case series, four case reports, and two cohort studies included in the final analysis (Table 1). The studies were performed in Europe (77%) and North America (13%). The total sample size of the included studies consisted of 345 patients, with 242 females (70%) and 103 males (30%). The mean age range was 30–80 years old. A total of 370 fractures were recorded, with 260 (70%) traumatic fractures and 110 (30%) pathologic fractures. The location of these fractures included 141 humeral fractures, 12 femoral fractures, 43 radius fractures, 29 ulna fractures, 43 hand fractures, 36 pelvic fractures, 4 tibia fractures, 36 fibula fractures, and 1 fracture of the sternum (Table 2). The mean ± standard deviation (SD) MINORS score was 12 ± 1.7.

**Table 1:** Characteristics of included studies in the final analysis

Authors	Publication year	Study design	Level of evidence	Sample size	Sex (M:F)	Age (mean)	Injury mechanism	Implant location (s)	MINORS score
Albertini et al. [15]	2020	Case report^a^	Level V	1	0:1	60	Pathologic	Upper extremity	–
Fourman et al. [8]	2020	Case report^a^	Level V	1	1:0	30	Pathologic	Femur	–
Gausepohl et al. [2]	2017	Retrospective case series	Level IV	132	26:106	72	Both	Upper extremity Lower extremity Pelvis	13
Hoellwarth et al. [9]	2020	Retrospective cohort	Level III	100	49:51	64	Pathologic	Humerus	13
Krumme et al. [6]	2021	Retrospective case series	Level IV	25	9:16	63	Pathologic	Upper extremity	14
Meijering et al. [16]	2018	Case report^a^	Level V	1	0:1	59	Pathologic	Femur	–
Oikonomidis et al. [14]	2019	Retrospective case series	Level IV	32	7:25	80	Trauma	Pelvis	12
Pesch et al. [17]	2019	Case report^a^	Level V	1	1:0	41	Pathologic	Femur	–
Surke et al. [12]	2020	Retrospective case series	Level IV	29	24:5	35	Trauma	Metacarpals	11
Van Oijen et al. [5]	2021	Retrospective case series	Level IV	26	1:25	77	Trauma	Distal radius	12
Vegt et al. [7]	2014	Retrospective case series	Level IV	33	8:25	77	Both	Upper extremity Lower extremity	9
Vegt et al. [1]	2018	Prospective cohort	Level IV	33	8:25	55-92^b^	Both	Humerus	13
Zoccali et al. [13]	2021	Retrospective case series	Level IV	12	7:5	66	Pathologic	Humerus	12

^a^Case reports were ineligible for MINORS risk of bias assessment

^b^Patient age was only reported as a range

**Table 2:** Anatomical location of fracture and PBSS

Fractures location	Number (n)
Humerus	141
Radius	43
Metacarpal	43
Pelvic rami	36
Fibula	35
Ulna	29
Femur	12
Tibia	4
Sternum	1

*PBSS* Photodynamic bone stabilization system

### Complications

One Level III [9], Eight Level IV [1, 2, 5–7, 12–14], and three Level V [8, 15, 16] studies reported complications following PBSS. A total of sixty complications were reported (Table 3). Post-operative non-device related complications comprised a majority of all complications (35; 58%). One intra-operative device failure (2%) was due to incomplete resin curing. The most common post-operative device-related complications were due to breakage of the implant (16; 27%), device dislocation (3; 5%), and implant protrusion (2; 3%).

**Table 3:** Intraoperative and postoperative implant complications

Complication	Number (n)
**Intraoperative: Device-related**	1
Incomplete resin curing	1
**Postoperative: Device-related**	24
Breakage of Implant	16
Implant protrusion	2
Device dislocation	3
Incorrect implant sizing	1
Implant misplacement	1
Periprosthetic fracture	1
Device related pain	1
**Postoperative: Non-device related**	35
Surgical site infections (SSI)	8
Neurapraxia	2
Complex regional pain syndrome	1
Persistent pain	2
Wound dehiscence	1
Decreased sensation	5
Skin adhesion	4
Delayed bone healing	2
Urinary tract infection (UTI)	4
Pneumonia	2
Improper screw positioning	1
Revision	1
Screw pull out	2

### Radiographic findings

Radiographic outcomes were reported by six Level IV studies and one Level V study (Table 4). Three studies[2, 5, 12] assessing fractures of the distal radius, metacarpals, humerus, and pelvis found complete fracture healing in all patients at 12-month follow up. Similarly, two studies [1, 14] found 96% fracture healing among pelvic and humerus fractures, respectively. One study [16] reported evidence of fracture displacement on an individual with osteogenesis imperfecta type 4. Lastly, one study reported adequate treatment of a pathologic humerus fracture in a patient with metastatic renal cancer.

**Table 4:** Radiographic outcomes following PBSS implantation

Authors	Fracture location	Radiographic key findings
Albertini et al. [15]	Humerus	• There was no secondary mobilization of the implants, an initial callus formation, and new ossification processes at two months
Gausepohl et al. [2]	Upper extremity Lower extremity Pelvis	• A total of 84% (21 of 25) of fractures had adequate reduction without residual deformity. Additionally, limb shortening was absent or < 5 mm for 88% (22 of 25) of fractures with 12 months of follow-up • Recurvatum/procurvatum deformity was absent in 80% (20 of 25) for fractures with 12 months of follow-up. There was no radiographic evidence of implant migration • Healing outcomes were excellent, with substantial cortical bridging and complete healing at 12 months, and total dissolution (100%) of the fracture line at the 3-month follow-up
Meijering et al. [16]	Femur	• Fracture fragments dislocated. Consolidation present at 6 months
Oikonomidis et al. [14]	Pelvis	• Consolidated pubis ramus fractures were reported in 96% of the cases at 6 months
Surke et al. [12]	Fifth metacarpal	• At a median follow-up of 13 weeks, all fractures had radiologically healed, and there were no instances of delayed union or non-union. The median shortening was 2 mm, and the median palmar angulation reduction was 31 degrees at all follow-ups
Van Oijen et al. [5]	Distal radius	• At follow-up, all radiographs showed appropriate bone healing without abnormalities, except for sclerosis around the implant. Volar angulation was present in 7 patients with a median of 5 degrees, while dorsal angulation was present in 23 patients with a median of 3 degrees • According to Lidstrom classifications, 6 patients had excellent outcomes, 17 had good outcomes, 5 had fair outcomes, and 2 had poor outcomes
Vegt et al. [1]	Humerus	• Complete radiographic fracture healing was observed in 81% (21/26, 95% CI (0.656, 1.000)) of patients with a valid assessment at 90 days, 88% (23/26, 95% CI (0.761, 1.000)) of patients at 180 days and 96% (27/28, 95% CI (0.895, 1.000)) at one year

*PBSS* Photodynamic bone stabilization system

Two studies [5, 12] reported angulation reduction after operation with the PBSS. Two studies [2, 12] found minimal limb shortening in a majority of their patients with one study [12] reporting a median of 2 mm shortening in fifth metacarpal fractures, while another study [2] found 86% of patients who had radiographic follow up having absent or < 5 mm shortening in their heterogeneous fracture cohort.

### Functional outcomes

Five Level IV and Two Level V studies reported functional outcomes (Table 4). Pain scores reported were overall minimal across studies. One study [5] found median pain scores of 0 at final follow up at rest and with activity. Three studies [7, 13, 14] utilized VAS scores to measure pain. VAS scores were consistently low in all studies, with one study [7] finding a significant decrease in VAS scores compared to baseline with up to 1 year of follow-up. DASH scores to measure disability were utilized in three studies [5, 7, 15] and demonstrated a significant downward trend of DASH scores reported until 1 year follow-up. Lastly, range of motion and strength were examined in five studies [1, 5, 7, 15, 17] which all found adequate range and strength recovery.

## Discussion

PBSS may be used as an alternative to intramedullary nailing and plates in the correction in the fixation of both traumatic fractures and pathologic/impending fractures. The aim of our systematic review was to report the indications, complications, and functional and radiographic outcomes following PBSS implantation. Our analysis found that the PBSS system was utilized as a treatment option for a range of injuries, including traumatic and pathologic fractures in the upper extremities, lower extremities, chest, and pelvis. PBSS was associated with a low incidence of complications, with only a minority of cases attributed to the device itself. The most common device-related complication was implant breakage. There was only one intra-operative complication reported due to incomplete resin curing. The majority of studies found complete fracture healing and low patient-reported pain and disability scores by 12 months. While these findings are encouraging, the majority of studies were retrospective case series, and future prospective randomized trials and comparative studies are necessary to evaluate the efficacy of PBSS.

### Complications

Our review of included studies showed a complication rate of 16% for the PBSS system, with 60% of these complications not related to the device. Among the device-related complications, breakage was the most common issue. Regarding device instillation, the PBSS had minimal intraoperative complications, with only one reported case of incomplete resin curing.

**Fig. 3:**
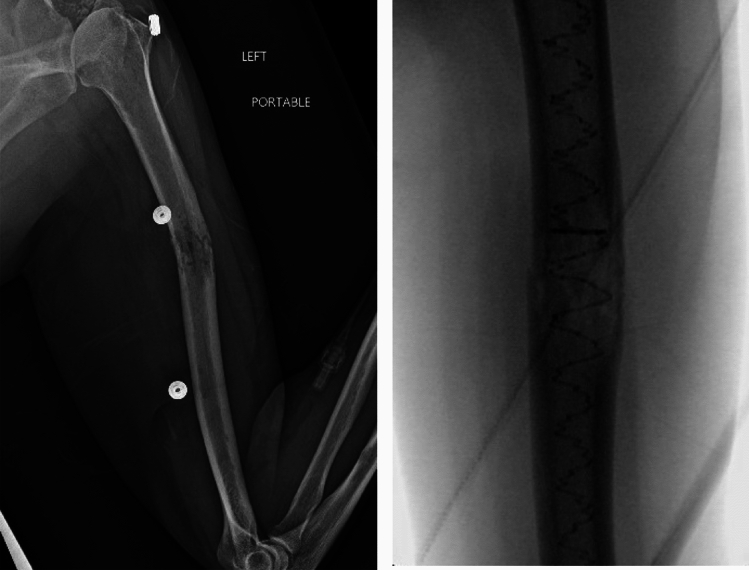
Example of a pathologic fracture of left humerus related to multiple myeloma stabilized with PBSS (i.e., IlluminOss) implant

**Fig. 4:**
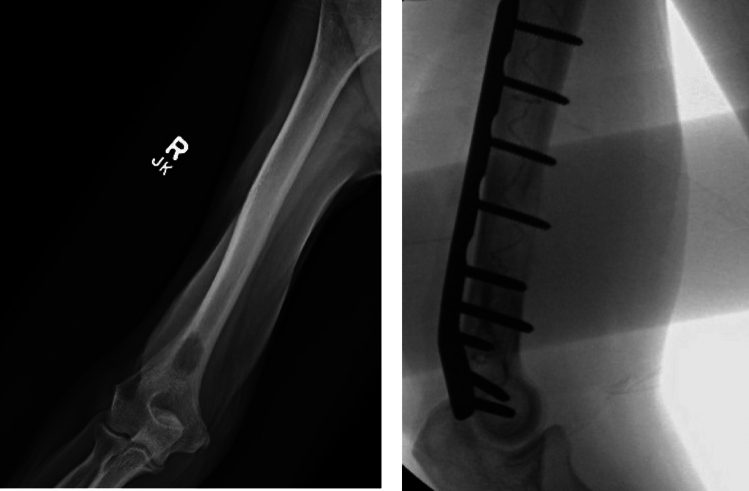
Metastatic bladder carcinoma with impending fracture of the distal humerus treated with PBSS (i.e., IlluminOss) augmented by plate

Compared to IMN and plate complication rates, PBSS offers a similar rate of overall complications. Distal radial fractures, for example, undergoing volar locking plates has a complication rate between 3 and 36% reported [18–20]. Similarly, among humeral shaft fractures, one study found complications of 58% for plating and 43% for IMN [21]. However, PBSS offers a number of benefits that may explain low rate of intra-operative complication rates. Our analysis found a majority of fractures treated with PBSS were humeral shaft fractures (Fig. 3). In the fixation of humeral fractures with IMN, a number of complications can occur such as protrusion of the nail can lead to pain and stiffness. Iatrogenic comminution can occur with previously reported rates of between 7 and 20%, and the diameter of the humeral canal may limit nailing efficiency. Furthermore, neurovascular injuries include risk to the radial nerve from the insertion of the nail, a risk to the axillary nerve from proximal locking, and a risk to the contents of the cubital fossa with distal locking [22]. The malleability of the intramedullary polymer in PBSS may provide an advantage over the limitations of plating fixation in terms of under or oversizing and misplacement [22]. PBSS can also offer stabilization with augmentation using a plate, which depends on the patient’s bone quality and density (Fig. 4). Patients with poorer quality of bone or lower density of bone may require this more comprehensive treatment.

### Radiographic findings

The use of PBSS in the treatment of fractures has been associated with favorable Radiologic outcomes, including almost complete fracture healing, minimal limb shortening, and no implant migration. Two studies demonstrated complete radiographic healing in all patients within three months following PBSS fixation [2]. Similarly, a study on humeral fractures treated with PBSS had a healing rate of 96% in one year. In comparison, a prospective study comparing IMN and plating for humeral shaft fractures found healing rates of 93 and 87%, respectively, at an average follow-up of 16 weeks [21]. Nonetheless, not all patients with PBSS fixation had complete fracture healing. One study found four patients with limb deformities, three patients with > 5 mm shortening, and Recurvatum/procurvatum deformity present in five individuals [2]. Lastly, two studies found volar and dorsal angular deformities present at follow-up [5, 7]. Further research is warranted to comprehensively assess the radiographic outcomes of PBSS across a range of fracture types and anatomic locations.

### Functional outcomes

The majority of studies found minimal pain scores with good recovery of function following PBSS. VAS scores were consistently low in all studies, with one study [7] finding a significant decrease in VAS scores compared to baseline with up to 1 year of follow-up. DASH scores to measure disability were utilized in three studies [5, 7, 15] and demonstrated a significant downward trend of DASH scores reported until 1 year follow-up. One study using PBSS for humeral shaft fracture reported a mean DASH score of 23.9 at one-year follow up. Similarly, a systematic review evaluating functional outcomes following locking plate fixation of humeral fractures found a similar DASH scores [23]. Lastly, the majority of studies found adequate range and strength recovery after 12 months. Given the reported functional findings thus far, PBSS seems to offer comparable functional outcomes compared to IMN and plating; however, research needs to directly compare these fixation modalities and their impact on functional recovery in patients with various types of fractures.

#### Cost effectiveness

As with any emerging novel technology, the economic effect of the PBSS should be discussed. In 2005, a model predicted the total number of fractures in the US to exceed 2 million and the economic cost to be $16.9 billion [24]. The model projected that number to increase to 3 million fractures per year at a cost of $25.3 billion in 2025. An analysis on economic burden among osteoporosis-related fracture in the Medicare US population found that the one of the highest costs included inpatient medical services and rehabilitation services, such as skilled nursing facilities [25]. One area that PBSS may have tangible effects on reducing healthcare cost and the economic burden on the healthcare system is decreasing inpatient hospital length of stay and expected faster recovery—which in turn, would lower costs of rehabilitation [26, 27]. However, there remains a need for an economic analysis on the impacts of the PBSS compared to traditional means of fracture repair to determine the effects of PBSS fixation on healthcare costs.

### Limitations

There are several limitations to consider. First, given that PBSS is a relatively recent technology, a limited amount of data is available, which restricted our analysis. Consequently, included studies often had varying inclusion criteria, resulting in significant heterogeneity among studies regarding the type and location of the fractures and the outcome data reported. This heterogeneity prevented us from conducting a meta-analysis, and the results were instead presented descriptively. Additionally, while we utilized a rigorous systematic review protocol (PRISMA), our analysis is susceptible to publication bias, as studies with negative or null results may not have been published. Therefore, caution must be taken when interpreting the results of this systematic review, and future studies must address these limitations to enhance our understanding of PBSS's optimal use and long-term outcomes. Lastly, there are very few studies that directly compare outcomes related to alternative methods of fracture fixation such as IMN and plating. Furthermore, no prospective, randomized control trials have assessed the efficacy of PBSS.

## Conclusion

The photodynamic bone stabilization system represents a promising option for fracture treatment across a range of fracture types, locations, and injury mechanisms. Both radiographic and functional outcomes have been favorable, with minimal device-related complications. While the existing literature primarily comprises non-comparative and retrospective studies, the results to date suggest that the PBSS has potential. More prospective randomized trials and comparative studies are necessary to evaluate the efficacy of PBSS relative to traditional fracture repair. Further research focuses on higher quality, randomized, and prospective data is required to fully understand the indications, optimal use, and long-term outcomes of PBSS.
